# A case at crossroads—urological presentation, cardiac complication and haematological diagnosis: should imaging be pursued prior to orchidectomy at all costs?

**DOI:** 10.1093/jscr/rjab177

**Published:** 2021-05-14

**Authors:** Zaid Yasen, Fawz Kazzazi, Kyriacos Ioannides, Shanti Velmurugan, Kris Zegocki, Chi Li

**Affiliations:** 1 Barts Health Trust, London, UK; 2 University of Edinburgh, Mason Institute of Life Sciences, Medicine & Law, Edinburgh UK; 3 University College London Hospitals, London, UK

## Abstract

This case report explores the interesting case of a 71-year-old gentleman who presented with a testicular lump following trauma. Ultrasound imaging of the testicle demonstrated malignancy and subsequently orchidectomy was listed. Due to a scheduling difficulty, this was prioritized ahead of his whole-body computed tomography scan. Intraoperatively, he developed electrocardiogram changes suggestive of a non-ST elevated myocardial infarction. Post-operative imaging demonstrated a diffuse large B-cell lymphoma encroaching the heart and greater vessels. This case report highlights the importance of preoperative imaging, even where it may prove challenging. We assess the adequacy of current guidelines within the UK on imaging for new testicular malignancies.

## INTRODUCTION

At present, guidance for imaging in patients with a new diagnosis of testicular malignancy on ultrasound imaging do not uniformly outline the chronology of further imaging and orchidectomy. Typically, a full-body computed tomography (CT) scan is sought prior to operation to explore the extent of further disease, particularly because post-operative survival rates are much lower in the presence of metastasis [1], and postponing the operation leads to delays in administration of chemotherapy.

Current guidelines outline conflicting orders for investigating new malignancy (Table 1**)**.

**Table 1:** 

Organization	Recommendation
NHS England [2]	An urgent CT-CAP should be booked once cancer confirmed on testicular ultrasound.
British Association of Urological Surgeons [3]	If cancer is confirmed, abdominal, chest and pelvic CT scanning is usually performed to stage the disease more accurately. It may, however, as a matter of **convenience**, be performed before orchidectomy.
Royal College of Radiologists [4]	Following orchidectomy and an established diagnosis of a testicular germ cell tumour, all patients should have initial staging with CT of CAP.
European Association of Urologists [5]	Contrast-enhanced CT is recommended in all patients for staging before orchidectomy but may be postponed until histopathological confirmation of malignancy.

In this case, scheduling challenges in the weeks pre-COVID meant that the operation went ahead prior to further imaging. Here, we explore details of the case and the consequences of this decision.

## CASE REPORT

**Figure 1:**
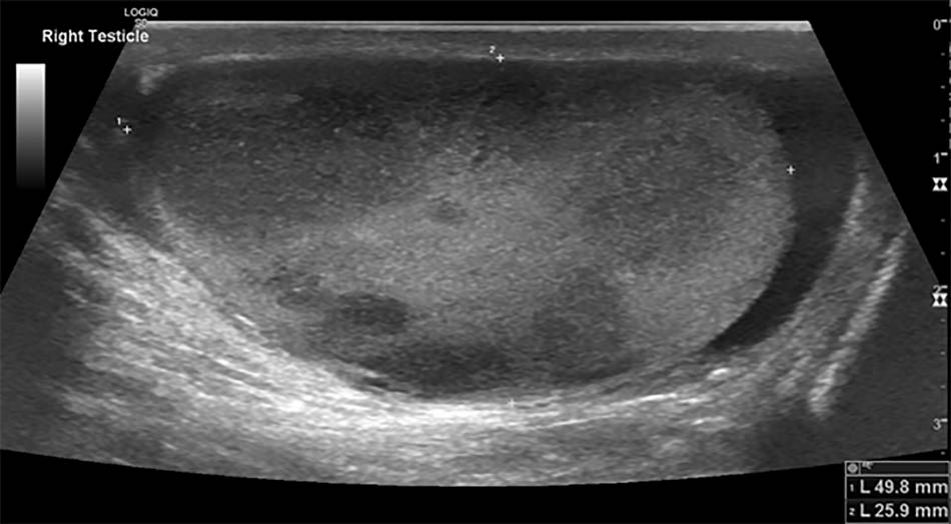
Testicular ultrasound of the right testicle demonstrating multiple hypoechoic lesions—the largest measuring 3 cm.

A 71-year-old man presented to the emergency department 3 days after falling from a ladder, having noticed a hard lump on his testicles. His past medical history included: osteoarthritis, hypercholesterolaemia and ‘prediabetes’; he had a family history of ischaemic heart disease. Examination revealed firm non-tender lumps on the right testicle, with no signs of testicular rupture. A subsequent ultrasound scan demonstrated ‘multiple hypoechoic lesions in the right testis (up to 3 cm) suspicious of malignancy’ (Fig. 1**)**. Tumour marker results demonstrated a raised lactate dehydrogenase at 426 but normal beta-human chorionic gonadotrophin and alpha-fetoprotein levels. His liver function tests revealed raised aspartate transaminase (62) and alkaline phosphatase (252) but normal bilirubin levels. The impression was a testicular lymphoma.

He was discussed at the multidisciplinary team (MDT) meeting, and due to scheduling clashes over the Christmas period, his operation was booked prior to CT staging. The following week, he underwent a right orchidectomy via inguinal approach as a day case and follow-up was planned with histology results and oncology MDT outcomes.

**Figure 2:**
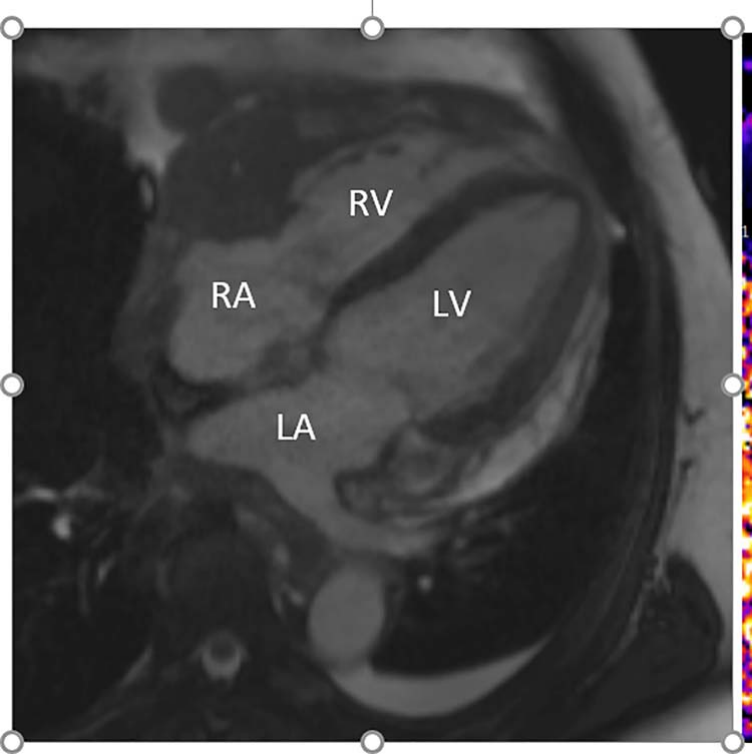
Cardiac MRI (transverse plane) demonstrating an enhancing lesion surrounding the chambers of the heart.

**Figure 3:**
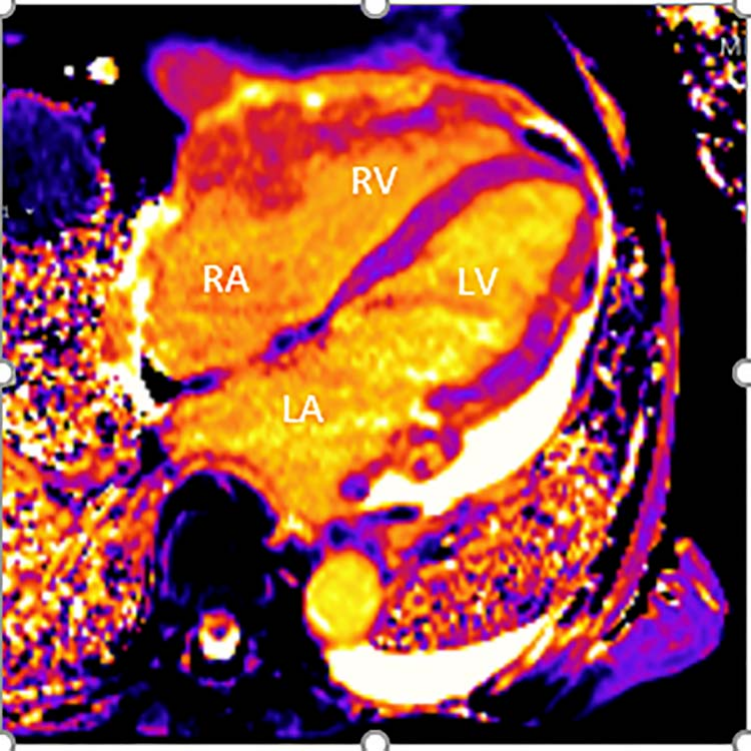
Cardiac MRI (transverse plane) demonstrating an enhancing lesion surrounding the chambers of the heart.

Intraoperatively, he developed new t-wave inversion on lead II of the 3-lead electrocardiogram (ECG). This resolved post-operatively, and he denied chest pain. He was kept in overnight for observation. The next morning, during the round, he reported central crushing chest pain the previous day that he had not disclosed. His ECG was repeated and demonstrated the t-wave inversion in lead II—indicating dynamic ECG changes. A troponin I level measured 27. As such, management for a non-ST elevation myocardial infarction was initiated and a cardiology consult organized, who advised continued acute coronary syndrome (ACS) treatment and an echocardiogram. This demonstrated a ‘pericardial mass (subcostal 3 cm × 2.5 cm) encroaching and extending outwards on both sides of the right atrioventricular groove’. ACS protocol was stopped and a cardiac magnetic resonance imaging (MRI) was organized (Figs 2 and 3**)**.

This demonstrated a bulky mediastinal tissue (measuring 50% of the heart) that encased all the aorta and pulmonary artery as well as the coronary artery. It has malignant behaviour with multiple foci that breached fascial planes. It restricted the long axis of the heart but was not causing haemodynamic occlusion. It was non-resectable.

The differential for this lesion included: primary tumour (angiosarcoma, lymphoma), metastatic tumour or (rarely) histiocytosis-type disease (Ehrdheim Chester/Rosai Dorfman). The working diagnosis remained lymphoma.

**Figure 4:**
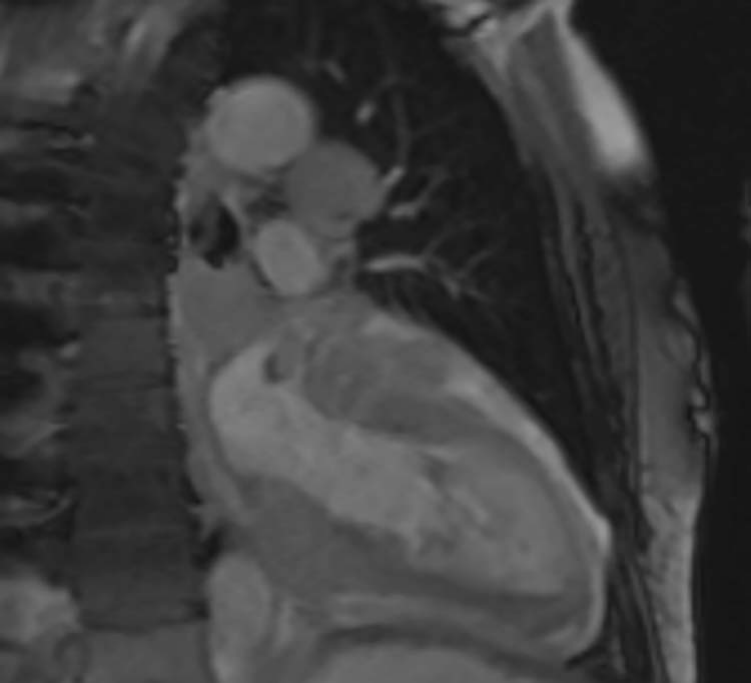
Cardiac MRI (sagittal plane) showing the lesion encasing the aorta, pulmonary artery and all coronary arteries of the heart.

**Figure 5:**
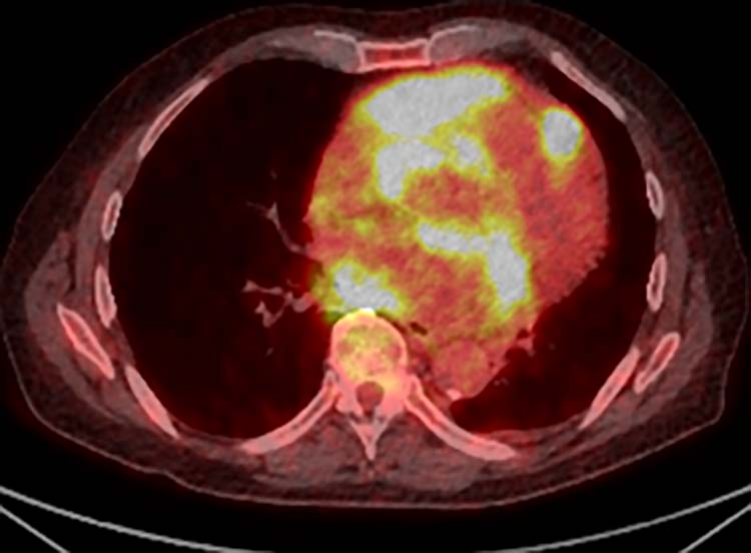
PET scan (transverse plane) demonstrating an enhancing lesion encroaching the heart with necrotic lymph nodes.

Further staging imaging was recommended by the radiology team. A CT head scan demonstrated no abnormalities, whereas CT chest–abdomen–pelvis (CAP) found significantly enlarged necrotic mediastinal lymph nodes with invasion into the pericardium and heart; bilateral adrenal nodules (suspicious for metastases) and appearances consistent with a lymphoproliferative disorder. The testicular biopsy found large lymphocytes with abundant cytoplasm and prominent nucleoli consistent with a diffuse large B-cell lymphoma (DLBCL). A positron emission tomography (PET) scan was organized at the request of the oncology team (Figs 4 and 5).

**Figure 6:**
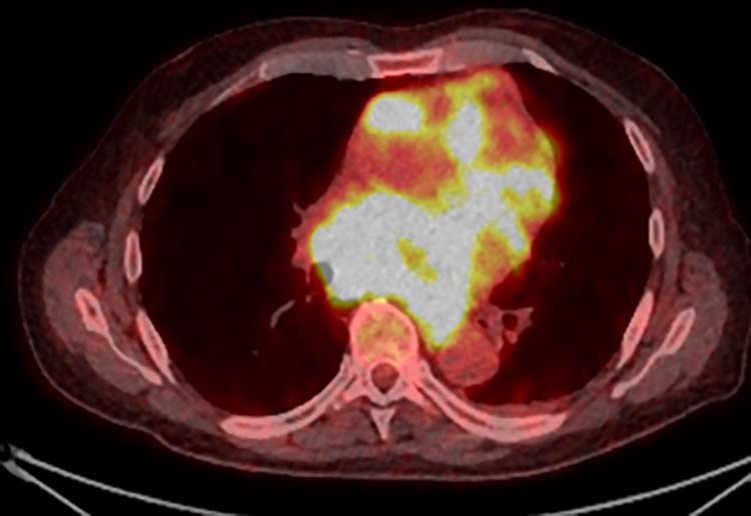
PET scan (transverse plane) demonstrating an enhancing lesion encroaching the heart with necrotic lymph nodes.

**Figure 7:**
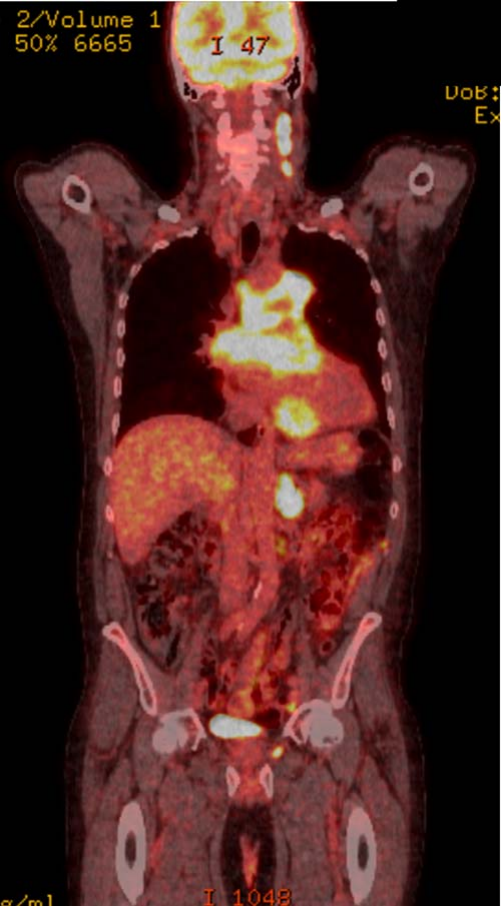
PET scan (coronal plane) showing intensely metabolically active lymphadenopathy on both sides with extra-nodal involvement through lymphoid tissue, adrenals and muscle.

He was subsequently diagnosed with stage IV (Lugano) DLBCL and underwent the R-CHOP chemotherapy regimen. He has responded well to his oncological treatment.

## DISCUSSION AND CONCLUSION

In men who present with a suspicious testicular lesion, it is important to perform a orchidectomy for a histological diagnosis [1]. Primary testicular lymphoma (PTL) is an uncommon (<5%) and aggressive form of extra-nodal non-Hodgkin lymphoma [6], and the most common cause of a new testicular lesion in men over the age of 60 [7]. DLBCL is the most common subtype—accounting for >75% [8]. On histological diagnosis of DLBCL, a PET–CT is recommended to determine the extent of disease [9]. In ~25% of these patients, there is secondary spread to the heart [10].

The urology team considered whether preoperative imaging and tissue biopsy would have altered this patient’s outcome; however, orchidectomy is the first-line management of patients with DLBCL presenting with primary or secondary testicular lesions [11]. Therefore, a tissue biopsy would not have influenced the decision to operate prior to imaging. However, there is no current consensus in the literature regarding the use of CT imaging before or immediately after orchidectomy [12], with the seminal work showing value in both instances dating back to the 1990s [12]. Staging his disease prior to the operation would have had implications for his anaesthetic assessment—particularly in determining cardiovascular risk. He did not undergo preoperative echocardiogram due to his high fitness level and functional status (Rockwood score 1).

This case demonstrates that, considering the increased incidence of DCBCL in patients over the age of 60, there can be significant clinical value in performing a CT scan before orchidectomy—in keeping with European guidance [5]. This is particularly important when considering the high incidence of DCBCL as the subtype of PTL—of which one-quarter have secondary spread to the heart. This suggests that all patients in whom PTL is the leading differential should undergo an echocardiogram prior to operation and, if positive, a staging CT scan.
